# Presentation, Treatment, and Prognosis of Secondary Melanoma within the Orbit

**DOI:** 10.3389/fonc.2017.00125

**Published:** 2017-06-23

**Authors:** Anna M. Rose, Sophie Cowen, Channa N. Jayasena, David H. Verity, Geoffrey E. Rose

**Affiliations:** ^1^UCL Institute of Ophthalmology, London, United Kingdom; ^2^Department of Medicine, Imperial College, London, United Kingdom; ^3^Orbital Unit, Moorfields Eye Hospital, London, United Kingdom

**Keywords:** melanoma, orbital tumors, orbital malignancy, orbital metastases, metastatic melanoma

## Abstract

**Background:**

Ocular melanoma is a rare but often deadly malignancy that arises in the uvea, conjunctiva, or orbit. Uveal melanoma is the most common type, with conjunctival melanoma being the second most frequently observed. Melanoma accounts for 5–10% of metastatic or secondary orbital malignancies, but only a minute proportion of primary orbital neoplasia. The aim of this study was to characterize the clinical presentation, treatment, and prognosis in patients presenting with melanoma metastatic to, or secondary within, the orbit.

**Methods:**

A retrospective cohort study of patients presenting to a tertiary referral orbital unit from 1982 to 2016 was performed. Eighty-nine patients with biopsy-proven diagnosis of melanoma within the orbit were included in the study. The clinical notes, radiological imaging, histology, surgical notes, and outcome data for the patients were reviewed. The main outcome measures of interest were the interval between primary malignant melanoma and orbital presentation, survival after orbital presentation, and clinical parameters (such as gender, age at presentation, and treatment approach).

**Results:**

The commonest primary source of tumor was choroidal melanoma, with conjunctival and cutaneous melanomas being relatively common; eyelid and naso-sinus tumors occurred in a few cases. The mean age at presentation with orbital disease was 65 years (31–97 years). The interval between primary malignancy and orbital disease (either local spread/recurrence or true metastatic disease) showed wide variability, with almost one-third of patients having orbital disease at the time of primary diagnosis, but others presenting many years later; indeed, the longest orbital disease-free interval was over 34 years. Twenty-three patients were considered to have had late orbital metastases—that is, at more than 36 months after primary tumor. The median survival following presentation with orbital involvement was 24 months. Patients with tumors of cutaneous origin had worst survival, whereas those with conjunctival tumors had the best prognosis.

**Conclusion:**

A high index of suspicion for orbital recurrence should be maintained in any patient with prior history of melanoma, however distant the primary tumor is in site or time. Furthermore, giving a prognosis for orbital melanoma remains problematic due to highly variable survival, and further investigation will be necessary to understand the likely genetic basis of this phenomenon.

## Introduction

Intraocular metastases from malignant melanomas usually affect sites favored by primary intraocular melanoma—namely, the uveal tract—but can also affect the optic disc, retina, and vitreous (1–5). Orbital metastases from any malignancy are rare, and metastatic melanoma accounts for 5–20% of these, with primary sites usually being the skin, uvea, or ocular surface (6–11). Late presentation of secondary orbital melanoma has occasionally been reported, with the longest disease-free interval being 40 years after enucleation for a choroidal melanoma (12), but in general, these patients have been reported to have very poor survival—the average being 5.7–19.7 months (10, 13, 14).

In this work, we extend the knowledge of the clinical presentation and therapeutic strategies for patients with secondary melanoma within the orbit, including patients with anatomically contiguous disease (e.g., local progression or local recurrence of ipsilateral ocular disease/sinus disease) and those with true metastatic disease (e.g., contralateral ocular disease or distant cutaneous sites). In particular, focus is placed on two unusual groups: first, those patients with a long interval between primary disease and orbital disease and, second, those with a long survival after treatment of melanoma within the orbit.

## Patients and Methods

Patients seen within the Orbital Unit at Moorfields Eye Hospital, between 1982 and 2016, with biopsy-proven orbital melanoma were identified from a diagnostic database, and the clinical case-notes and imaging were reviewed (where available). Patients with proven primary orbital melanoma (e.g., *without* evidence of ipsilateral intraocular, ocular surface, or sinus disease) were excluded. For this study, “late” orbital recurrence was regarded as being 3 or more years after treatment of the primary tumor, and “long survival” was 4 or more years after diagnosis of secondary orbital disease. The study was conducted in accordance with the Declaration of Helsinki. All participants had given written, informed consent for inclusion of data in studies prior to surgery or treatment. Local ethics board (Moorfields Eye Hospital NHS Foundation Trust) approved the study under the retrospective analysis of data procedures (ROAD17/010).

Survival analysis was estimated using standard Kaplan–Meier survival plots (on MedCalc); normality of data was assessed using Shapiro–Wilks testing, and comparison of means was performed using Mann–Whitney *U*-testing for non-parametric data.

## Results

**Figure 1:**
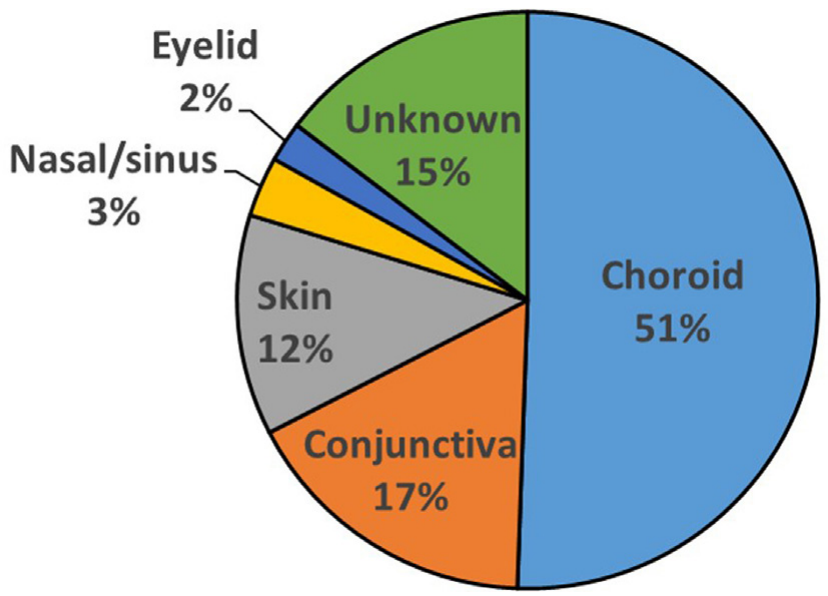
Sites of primary origin for malignant melanomas for 89 patients presenting with melanoma in the orbit.

Eighty-nine patients were identified with secondary orbital melanoma during the study period, with complete clinical data available for 48 (54%) patients (Table 1), and partial data—including mortality data—for a further 21 patients (24%) (Table 2); only very limited data were available for 20 patients. The commonest primary source of tumor was choroidal melanoma (45/89; 51%); conjunctival melanoma was the next most frequent (15/89; 17%), and cutaneous, eyelid, and naso-sinus tumors occurred in a few cases; the primary origin could not be ascertained in 11/89 (12%) cases due to disseminated disease at presentation (Figure 1). The mean age at presentation with secondary orbital disease was 65 years (median 63; range 31–97 years). There was no significant gender bias, with 50 affected women and 39 men (χ^2^ = 2.72, *p* > 0.05); this lack of gender bias persisted after being stratified by the decade of presentation (*p* > 0.05). Most patients were white northern Europeans, but four were of Mediterranean origin, one from the Middle East, and one from North Africa.

**Table 1:** Clinical characteristics of 48 patients with orbital malignant melanoma secondary to a primary tumor elsewhere, classified by the site of primary tumor and the survival interval after presentation with orbital disease.

Sex	Site of primary tumor	Age at presentation with primary tumor	Therapy for primary tumor	Age at secondary orbital disease (years)	Interval between primary therapy and orbital presentation (months)	Side	Treatment for secondary orbital disease	Orbital progression	Systemic progression	Interval between primary therapy and latest follow-up (months)	Survival after orbital treatment (months)	Age at death (years)
M	Choroid	77	Enucleation	79	32	L	Palliation			34	2	79
F	Choroid	89	Incisional biopsy	89	0	L	Incisional biopsy	N	Y	3	3	89
F	Choroid	73	Incisional biopsy	73	0	L	Incisional biopsy + RT	N	N	14	14	75
M	Choroid	49	Local resection	72	282	L	Debulking + chemotherapy	Y	Y	299	17	73
M	Choroid	47	Enucleation	57	114	R	Radiotherapy			138	24	58
F	Choroid	69	Enucleation	69	6	R	Exenteration	N	Y	30	24	71
F	Choroid	68	Exenteration + RT	68	0	R	(Exenteration + RT)	U	U	24	24	70
F	Choroid	67	Enucleation + RT	67	1	L	Debulking	Y	N	26	25	69
M	Choroid	37	Refused therapy	41	49	R	Exenteration + RT	Y	Y	84	35	43
F	Choroid	72	Palliation	72	0	L	(Palliation)	N	Y	39	39	75
F	Choroid	53	Enucleation	54	11	R	Exenteration + RT	N	Y	53	42	57
F	Choroid	59	Exenteration	59	0	R	(Exenteration)	N	Y	56	56	63
M	Choroid	23	RT	44	262	L	Debulking + RT	Y	Y	322	60	49
M	Choroid	71	Enucleation	71	0	R	(Enucleation)	N	N	105	105	80
M	Choroid	63	Exenteration + RT	63	0	R	(Exenteration + RT)	N	N	134	134	74
M	Choroid	60	Enucleation	62	25	L	Exenteration	N	N	184	159	75
M	Choroid	5	Enucleation	39	416	R	Exenteration	N	N	814	398	72
F	Choroid	62	Exenteration + RT	62	0	R	(Exenteration + RT)	N	N	17	17	N/A
F	Choroid	41	Exenteration + RT	41	0	L	Exenteration + RT	N	Y	30	30	N/A
M	Choroid	41	Enucleation	60	239	R	Debulking + RT	N	N	412	173	N/A
F	Choroid	36	Enucleation	53	208	R	Excisional biopsy + RT	Y	N	398	190	N/A
M	Choroid	48	Enucleation	49	16	L	Exenteration	N	Y		Lost to follow-up	Died date unknown
F	Choroid	54	RT	58	56	L	Exenteration + RT	N	N		Lost to follow-up	Died date unknown
F	Conj	70	Exenteration	70	0	R	Exenteration	N	Y	2	2	70
F	Conj	82	Excisional biopsy	82	8	R	Exenteration	U	Y	46	38	71
M	Conj	55	Local resection	56	12	R	Excisional biopsy	Y	Y	208	196	73
F	Conj	87	Local resection + RT	97	120	L	Exenteration	N	N	124	4	N/A
M	Conj	69	Local resection + RT	75	80	R	Exenteration + chemotherapy	N	Y	87	7	N/A
M	Conj	65	Local resection	69	51	R	Excisional biopsy	N	N	70	29	N/A
F	Conj	48	Local resection	59	128	R	Exenteration	N	Y	164	36	N/A
F	Conj	65	Local resection + RT	65	11	L	Debulking	Y	Y	63	52	N/A
M	Conj	87	Exenteration	87	0	R	Exenteration	N	Y	59	59	N/A
F	Conj	60	Nil	61	6	L	Exenteration	N	N	246	240	N/A
M	Skin	36	Local resection	43	87	L	Debulking + chemotherapy	N	Y	89	2	43
F	Skin	44	Local resection	63	234	L	Excisional biopsy	N	Y	240	6	64
F	Skin	58	Palliation	58	0	R	Palliation	N	Y	8	8	58
M	Skin	86	Local resection	88	28	R	Palliation	Y	Y	36	8	88
F	Skin	51	Local resection	57	83	L	Incisional biopsy + chemotherapy	N	Y	96	13	59
M	Skin	31	Local resection	34	52	BOTH	Extensive resection	U	U	72	20	36
M	Skin	51	Local resection	60	121	R	Biopsy + chemotherapy	N	Y	142	21	62
F	Skin	32	Local resection	64	362	L	Exenteration	N	N	363	1	N/A
F	Skin	54	Local resection	60	79	L	Exenteration + RT	N	N	87	8	N/A
M	Skin	54	Local resection	71	205	L	Debulking		Y	295	90	N/A
F	Skin	65	Local resection	67	41	R	Debulking + RT	N	Y	191	150	N/A
M	Unknown	61	Palliation	61	0	R	Palliation	N	Y	4	4	61
F	Nasal	54	Local resection	67	161	L	Debulking	Y	Y	201	45	71
F	Nasal	57	Local resection	61	48	L	Debulking + RT	U	Y	101	53	64
F	Sinus	59	Local resection	59	1	L	Debulking + RT	N	N	100	99	N/A

*RT, radiotherapy*.

*Gray shaded cases can be considered truly metastatic disease, whilst non-shaded cases are likely to represent local spread or local micrometastasis*.

**Table 2:** Survival after presentation with secondary orbital melanoma in 21 patients for whom date of death is known, without other complete clinical data; classified by origin of the primary tumor.

Sex	Age at orbital presentation (years)	Site of primary melanoma	Age at death (years)	Survival after presentation with secondary orbital disease (months)
M	57	Choroid	58	8
F	82	Choroid	83	20
M	64	Choroid	64	20
M	63	Choroid	66	38
M	54	Choroid	57	42
F	86	Choroid	91	66
F	75	Choroid	85	125
F	47	Choroid	74	328
M	63	Conjunctiva	64	21
F	81	Conjunctiva	83	32
F	60	Conjunctiva	65	63
M	78	Conjunctiva	90	140
F	64	Nasal	64	1
M	63	Sinus	63	3
F	53	Unknown	53	3
M	61	Unknown	60	5
F	62	Unknown	64	25
F	75	Unknown	78	50
F	63	Unknown	69	70
F	67	Unknown	73	73
M	87	Unknown	70	89

*Patients classified as having “unknown” origin for their primary tumor had extensive systemic disease at presentation and poorly differentiated histology, this precluding a conclusive diagnosis of the primary site*.

**Figure 2:**
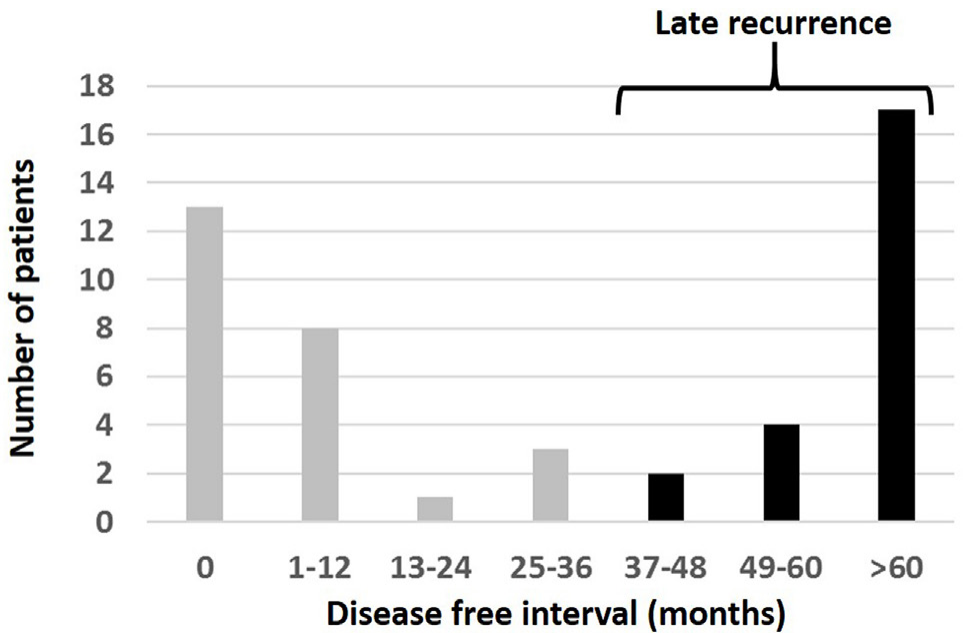
Interval from the time of diagnosis of primary melanoma to the appearance of orbital disease in 48 patients presenting with melanoma in the orbit. Patients were considered to have “late recurrence” when orbital disease presented more than 3 years after the primary diagnosis elsewhere.

The interval between the diagnosis of primary tumor and the later detection of orbital disease (the “orbital disease-free interval”) was available for 48/89 (54%) patients. This showed a wide variation, with 13/48 (27%) patients having orbital disease found at the time of primary diagnosis, but others presenting many years later; indeed, the longest orbital disease-free interval was over 34 years (Table 1; Figure 2). Twenty-three (23/48; 48%) patients were considered to have had late orbital disease—that is, at more than 36 months after primary tumor—and 17/23 (74% of the late recurring tumors) had very late recurrence (>6 years after primary disease). The commonest primary origin for late recurrent orbital tumors was skin (9/23 cases; 39%) or uveal tract (8/23; 35%).

**Figure 3:**
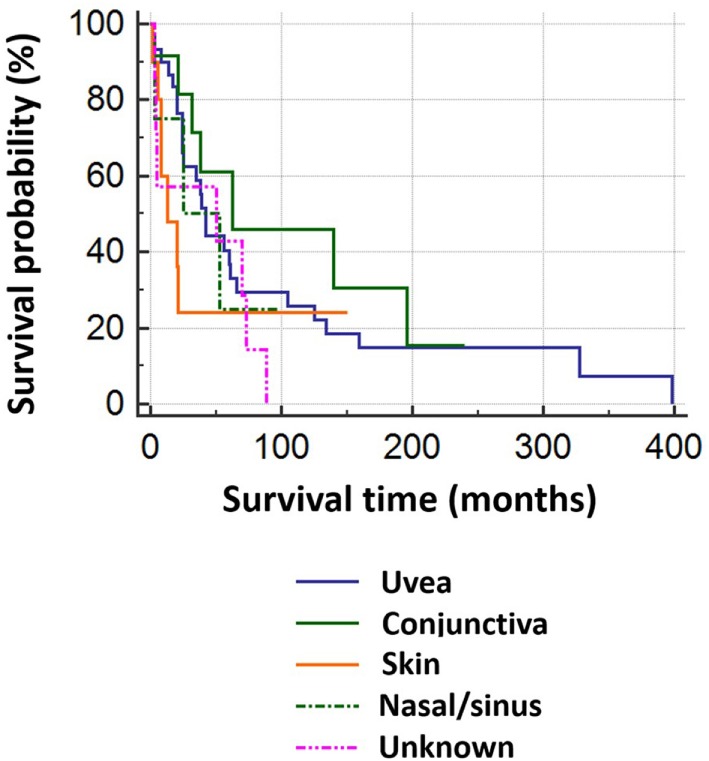
Kaplan–Meier survival analysis for 69 patients with orbital melanoma secondary to primary disease at another site, classified by origin of the primary tumor.

**Figure 4:**
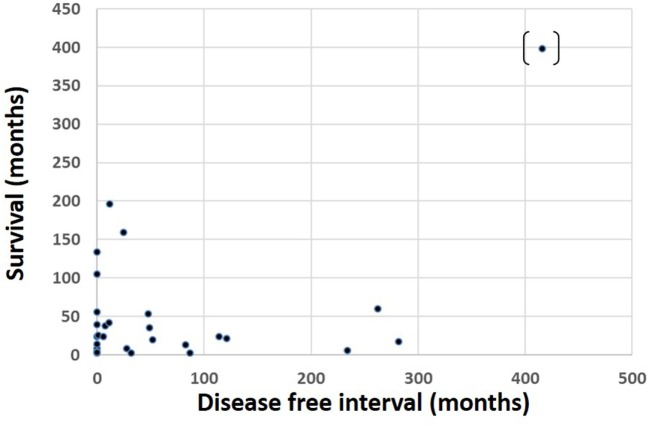
Survival time after presentation with orbital disease in 69 patients, as related to the interval between primary tumor diagnosis and orbital presentation.

Of 69 patients whose mortality data were available in May 2016, 52 had died (47/52 known tumor-related deaths) at a median of 24 months after orbital diagnosis (range 2 months to 33.2 years). Patients with tumors of cutaneous or unknown origin had worst survival, conjunctival had the best, and choroidal had the second best survival (Figure 3). There was no correlation between the orbital disease-free interval and overall survival (r = –0.16; one outlier value excluded) (Figure 4). The overall survival after orbital disease patients was similar in patients with early and late orbital recurrence (*U* = 162, *Z* = −0.399, *p* = 0.689); this latter analysis was performed excluding any patients presenting after May 2012 to avoid bias favoring the “early-recurring” group. Furthermore, the patients with locally progressive or locally recurrent secondary disease had similar survival to that of the patients with metastatic secondary disease (*U* = 97.5, *Z* = 0.400, *p* = 0.690). The median follow-up for the 17 known living patients is 44 months (range 1–190 months), suggesting a relatively good survival in this group.

Sixteen patients survived 4 years or more after orbital diagnosis (range 52–398 months), and eight are still alive (surviving to-date 52–190 months); the primary tumor was from choroid (eight cases), conjunctiva (four cases), skin (two cases), or sinus (two cases).

### Surgical Approaches in Secondary Orbital Melanoma

Where imaging showed diffuse orbital disease, an incisional biopsy was performed without any mobilization of bone, and, where orbital disease was causing major disfigurement, the patient was later considered for palliative orbital exenteration. However, in many cases, secondary orbital melanoma formed a well-defined mass at the time of presentation, and, in such cases, the mass was excised intact through a bone-sparing anterior orbitotomy. Where macroscopically intact excision was not possible, the lesion was, as far as possible, meticulously isolated from the “normal” neighboring tissues, and then piecemeal excision of the mass (debulking) was performed.

## Discussion

A retrospective analysis has been performed for 89 patients presenting with biopsy-proven secondary orbital melanoma in a tertiary-referral orbital unit; this analysis included two closely related classifications of secondary orbital of tumor—those that were locally progressive/recurrent and those that had metastasized from distant sites. There were two notable features in the patient group: first, orbital involvement tends to occur either early (<1 year after primary disease) or late at many years after primary disease (Figure 2). Second, there was a group of patients with an unusually long survival after the diagnosis of melanoma recurrence within the orbit.

Thirteen patients had orbital melanoma as the presenting symptom of a primary melanoma arising elsewhere; 11 of these were—somewhat predictably—ocular primary tumors with local disease progression; one primary tumor was, however, a distant cutaneous lesion, and one patient had widespread systemic disease and thus primary origin was not discernible. Late recurrence of choroidal melanoma is, however, fairly common—with 9% of recurrences occurring at 5–10 years after treatment (15). Of our 48 patients with complete follow-up, the time from initial diagnosis to presentation with orbital disease varied from 0 months to >34 years; remarkably, 23/48 (48%) had late secondary local recurrence, with an interval of 36 months or more between primary disease and orbital recurrence. This propensity for late orbital recurrence might, therefore, suggest that a disease-free interval of 10 years should not be considered a definite “cure” for malignant melanoma.

The reason for the long interval between primary disease and orbital recurrence in our patients is conjectural, but it might be related to immune-mediated control of tumor growth. Tumor-specific lymphocytes that target malignant cells through recognition of tumor-associated antigens—such as tyrosinase, Melan-A/MART-1, gp100, TRP-1, and TRP-2—have been isolated from melanoma (16). Furthermore, melanomas showing spontaneous regression contain more tumor-specific lymphocytes than those in non-regressing tumors, and the presence of these lymphocytes portends a better prognosis (17, 18). High levels of immunosurveillance within the orbit might, therefore, maintain micro-metastases of primary tumor in remission for many years; later, mutations might occur in tumor-associated antigens, leading to loss of lymphocytic recognition and, thereby, loss of tumor control. Alternatively, detrimental changes in the immune system (such as pregnancy, immunocompromise, or aging) might lead to reduced efficiency of immune surveillance, with emergence of growth in previously well-controlled micro-metastases.

Although choroidal melanoma was the commonest primary source for orbital melanoma, cutaneous melanoma is the most likely to have late orbital secondary disease. In a previous study of over 500 metastatic skin cancers, early metastasis (<3 years after surgery) was found to be significantly associated with past history of non-melanoma skin cancer, thicker lesions (Breslow depth), and ulcerative melanomas (19). In contrast, a history of *non-ulcerative* melanoma was found to be associated with late recurrence (>8 years after primary disease), this being consistent with our observation of tendency for orbital metastases of cutaneous melanoma to be a late phenomenon.

Published survival rates for melanoma within the orbit range from 6 to 20 months (10, 13, 14). There were 52 deaths (47 tumor-related) among our 69 patients with known outcome (75%), but only 11/52 (21%) fell within this previously published survival range, and 7/52 (13%) had less than 6 months survival. Moreover, 34/52 (65%) patients survived >20 months, and 19/34 (55.8%) survived >4 years. Of the 17 surviving patients in May 2016, 8 (47%) have survived more than 4 years. Our study would suggest that secondary malignant melanoma within the orbit can follow a relatively indolent course after treatment, with a survival much better than expected—thus making prognosis for this condition hard to predict.

Treatment of secondary orbital melanoma remains controversial and is often palliative (20). Surgery remains the mainstay of treatment, with local resection, debulking, or exenteration being the primary choices. Radiotherapy and chemotherapy can be considered, and their use takes into account life expectancy and the presence of other metastatic diseases (e.g., bony metastases). Local radiotherapy is the most commonly used adjuvant therapy when there are no distant metastases (10), and 16/48 of our patients had local radiotherapy. Chemotherapy was used in 4/48 (9%) of our patients and played a role when there is systemic metastatic disease at orbital presentation (e.g., bone or liver lesions). The scenario for melanoma chemotherapy is, however, changing rapidly with the advent of immunotherapy; cutaneous and uveal melanomas are biologically distinct, and, as such, they would be expected to require distinct treatments. Immunotherapy for metastatic cutaneous melanoma markedly improves survival, with the most dramatic being ipilimumab and nivolumab combination therapy (21). Several agents have been approved by FDA and NICE for monotherapy, while combination therapies undergo Phase III trials, but there is no evidence yet for the impact these may have in orbital metastases—a potential area for further research.

By contrast, ipilimumab has demonstrated only modest benefits in treating primary uveal melanoma. There are a variety of new agents that might provide benefit in metastatic uveal melanoma; these include verteporfin (previously used in wet age-related macular degeneration), arylsulfonamides, and anti-VEGF agents (22). Arylsulfonamides such as KCN1 inhibit hypoxia-inducible factors, thereby depriving cancer cells of their ability to thrive in a hypoxic environment (23). Anti-VEGF agents such as bevacizumab have been postulated as possible treatments because of the high VEGF levels seen in uveal melanoma. Clinical trials relating to these are all in early human phases but have demonstrated significant benefits in mouse models. These potential treatments of secondary orbital melanoma require further investigation, but they may herald a new era where metastatic melanoma is no longer a life-ending diagnosis.

In summary, a high index of suspicion for orbital recurrence should be maintained in any patient with prior history of melanoma, however distant the primary tumor is in site or time. Furthermore, giving a prognosis for orbital melanoma remains problematic due to highly variable survival, and further investigation will be necessary to understand the likely genetic basis of this phenomenon. Currently, surgery remains the mainstay of therapy in melanoma, but development of new immunotherapeutic agents might revolutionize therapy in the years to come.

## Ethics Statement

The local ethics committee ruled that the study did not require ethics approval for retrospective case note review (ROAD17/010).

## Author Contributions

AR—study design, data collection, data analysis, preparation of manuscript. SC—data analysis, preparation of manuscript. CJ—data analysis, revision of manuscript. DV—clinical assessment of patients, revision of manuscript. GR—study design, clinical assessment of patients, data analysis, revision of manuscript.

## Conflict of Interest Statement

The authors declare that the research was conducted in the absence of any commercial or financial relationships that could be construed as a potential conflict of interest.

## References

[1] de Bustros S Augsburger JJ Shields JA Shakin EP Pryor CC II. Intraocular metastases from cutaneous malignant melanoma. Arch Ophthalmol (1985) 103:937–40. 10.1001/archopht.1985.01050070063031 4015484

[2] Font RL Naumann G Zimmerman LE Primary malignant melanoma of the skin metastatic to the eye and orbit: report of ten cases and review of the literature. Am J Ophthalmol (1967) 63:738–54. 10.1016/0002-9394(67)91300-1 6022246

[3] Gündüz K Shields JA Shields CL Eagle RC Jr. Cutaneous melanoma metastatic to the vitreous cavity. Ophthalmology (1998) 105:600–5. 10.1016/S0161-6420(98)94011-8 9544631

[4] Young SE Retinal metastases. In: Ryan SJ, editor. Retina. St. Louis: Mosby (1989). p. 591–6.

[5] Ramaesh K Marshall JW Wharton SB Dhillon B. Intraocular metastases of cutaneous malignant melanoma: a case report and review of the literature. Eye (1999) 13:247–50. 10.1038/eye.1999.60 10450391

[6] Valenzuela AA Archibald CW Fleming B Ong L O’Donnell B Crompton J Orbital metastasis: clinical features, management and outcome. Orbit (2009) 28:153–9. 10.1080/01676830902897470 19839900

[7] Shields CL Shields JA Peggs M Tumours metastatic to the orbit. Ophthal Plast Reconstr Surg (1988) 4(2):73–80. 10.1097/00002341-198804020-00003 3154725

[8] Shields JA Shields CL Brotman HK Carvalho C Perez N Eagle RC Jr. Cancer metastatic to the orbit: the 2000 Robert M. Curts Lecture. Ophthal Plast Reconstr Surg (2001) 17(5):346–54. 10.1097/00002341-200109000-00009 11642491

[9] Goldberg RA Rootman J Cline RA Tumours metastatic to the orbit: a changing picture. Surv Ophthalmol (1990) 35(1):1–24. 10.1016/0039-6257(90)90045-W 2204127

[10] Zografos L Ducrey N Beati D Schalenbourg A Spahn B Balmer A Metastatic melanoma in the eye and orbit. Ophthalmology (2003) 110(11):2245–56. 10.1016/j.ophtha.2003.05.004 14597536

[11] Ahmad SM Esmaeli B Metastatic tumours of the orbit and ocular adnexa. Curr Opin Ophthalmol (2007) 18(5):405–13. 10.1097/ICU.0b013e3282c5077c 17700235

[12] Coupland SE Sidiki S Clark BJ McClaren K Kyle P Lee WR Metastatic choroidal melanoma to the contralateral orbit 40 years after enucleation. Arch Ophthalmol (1997) 115(1):134 10.1001/archopht.1997.01100150136036 9006447

[13] Orcutt JC Char DH. Melanoma metastatic to the orbit. Ophthalmology (1988) 95:1033–7. 10.1016/S0161-6420(88)33061-7 3231441

[14] Goldberg RA Rootman J Clinical characteristics of metastatic orbital tumours. Ophthalmology (1990) 97:620–4. 10.1016/S0161-6420(90)32534-4 2342807

[15] Diener-West M Reynolds SM Agugliaro DJ Caldwell R Cumming K Earle JD Development of metastatic disease after enrolment in the COMS trials for treatment of choroidal melanoma: Collaborative Ocular Melanoma Study Group Report No. 26. Arch Ophthalmol (2005) 123(12):1639–43. 10.1001/archopht.123.12.1639 16344433

[16] Yaeger T Brady L Basis for current major therapies for cancer. 1st ed In: Lenhard RE Jr Osteen RJ Gansler T, editors. Clinical Oncology. (Chap. 5), Atlanta: American Cancer Society (2000). p. 159–229.

[17] Connolly G Wladis E Masselam K Weinberg DA. Contralateral orbital melanoma 28 years following enucleation for choroidal melanoma. Orbit (2007) 26:291–4. 10.1080/01676830601169189 18097971

[18] Halliday GM Patel A Hunt MJ Tefany FJ Barnetson RS Spontaneous regression of human melanoma/non-melanoma skin cancer: association with infiltrating CD4+ T cells. World J Surg (1995) 19:352–8. 10.1007/BF00299157 7638987

[19] Brauer JA Wriston CC Troxel AB Elenitsas R Shin DB Guerry D Characteristics associated with early and late melanoma metastases. Cancer (2010) 116:415–23. 10.1002/cncr.24724 19918926

[20] Polito E Leccisotti A. Primary and secondary orbital melanomas: a clinical and prognostic study. Ophthal Plast Reconstr Surg (1995) 11:169–81. 10.1097/00002341-199509000-00003 8541258

[21] Larkin J Chiarion-Sileni V Gonzalez R Grob JJ Cowey CL Lao CD Combined nivolumab and ipilimumab or monotherapy in untreated melanoma. N Engl J Med (2015) 373(1):23–34. 10.1056/NEJMoa1504030 26027431 PMC5698905

[22] Luke JJ Triozzi PL McKenna KC Van Meir EG Gershenwald JE Bastian BC Biology of advanced uveal melanoma and next steps for clinical therapeutics. Pigment Cell Melanoma Res (2015) 28(2):135–47. 10.1111/pcmr.12304 25113308 PMC4326637

[23] Burroughs SK Kaluz S Wang D Wang K Van Meir EG Wang B. Hypoxia inducible factor pathway inhibitors as anticancer therapeutics. Future Med Chem (2013) 5(5):553–72. 10.4155/fmc.13.17 23573973 PMC3871878

